# Development and Psychometric Validation of the Cyprus Aphasia Screening Test (CAST)

**DOI:** 10.3390/brainsci16010032

**Published:** 2025-12-25

**Authors:** Marina Charalambous, Phivos Phylactou, Maria Kambanaros

**Affiliations:** 1 Department of Rehabilitation Sciences, School of Health Sciences, Cyprus University of Technology, Limassol 3036, Cyprus; pphylactou@unr.edu (P.P.); maria.kambanaros@cut.ac.cy (M.K.); 2 Cyprus Stroke Association, Limassol 3026, Cyprus; 3 Department of Psychology, University of Nevada, Reno, NV 89557, USA

**Keywords:** aphasia, screening test, psychometric testing, stroke

## Abstract

**Background/Objectives**: Aphasia screening tools help healthcare professionals detect aphasia after a stroke. To date, there is no standardized and validated aphasia screening tool available for use in Cyprus. The Cyprus Aphasia Screening Test (CAST) is a newly developed tool for detecting post-stroke aphasia. This study aims to present the main characteristics of the CAST and evaluate its psychometric properties and diagnostic accuracy. **Methods**: A cross-sectional study was conducted, involving 99 participants divided into three groups as follows: 43 people with stroke-aphasia, 21 with strokes but without aphasia, and 35 healthy controls. **Results**: The CAST demonstrated excellent internal consistency (Cronbach’s α > 0.967), high test–retest (ICC ≥ 0.983) and interrater (ICC = 0.979) reliability, and verified known-groups validity (*p*< 0.001). A significant correlation between the total scores of the CAST and the Greek version of the Boston Diagnostic Aphasia Examination (Short Form) confirmed a linear relationship across the two measures (*p* < 0.001). A ROC curve analysis (AUC = 0.97) identified 36/40 as the cut-off for detecting aphasia. **Conclusions**: The CAST is a reliable, clinician-administered aphasia screening tool with strong psychometric properties. It is designed to identify post-stroke aphasia and distinguish between stroke patients with and without aphasia. It consists of 10 subtests that assess both language comprehension and production. The CAST is designed for easy scoring and requires minimal equipment, making it well-suited for quick and efficient administration at the bedside. The CAST represents a step forward in aphasia screening for Greek-speaking populations in Cyprus.

## 1. Introduction

Around 12 million new strokes happen globally every year [1]. Of these, approximately 1.12 million new strokes occur in Europe, with 9.53 million stroke survivors and 7.06 million Disability-Adjusted Life Years (DALYs) lost due to stroke [2]. In Cyprus, an estimated 2300 new strokes occur annually, with approximately 19,600 people living with stroke-related consequences and around 14,500 DALYs lost each year [2].

Aphasia, an acquired language disorder caused by stroke, impairs a person’s ability to understand and/or produce language [3]. Based on established prevalence rates, approximately 40% of acute-phase stroke patients are expected to present with aphasia [4], meaning that around 920 new cases of aphasia occur each year in Cyprus, with 25–30% continuing to experience aphasia in the subacute phase and 15–20% individuals living with chronic aphasia [5]. Over time, people with aphasia experience greater disability than stroke survivors without aphasia and are less likely to regain independence or return to their daily routines, often requiring more intensive rehabilitation support [6]. Given its significant impact on communication and quality of life, early and accurate bedside detection of aphasia is essential to optimize recovery outcomes [7].

Screening tools are primarily designed for rapid detection of aphasia and referral decisions and are not typically equipped to assess the severity of language impairment [8]. These tools are administered in a few minutes and usually do not require specialized training, allowing a wide range of healthcare professionals, including physicians, nurses, and rehabilitation clinicians, to use them at the bedside [9]. In Cyprus, aphasia screening is often carried out by speech and language therapists (SLTs) and physicians using informal or ad hoc translations of English-language screening tools. These practices undermine the reliability and validity of the result, leading to inconsistent identification of aphasia, reduced diagnostic accuracy, and potential delays in initiating formal SLT assessments [6].

### 1.1. International Aphasia Screening Tests and the Need for Culturally Relevant Tools

A recent systematic review identified a wide range of aphasia screening tools used internationally [10]. Most available tools originate from English-speaking contexts, with translations subsequently produced for other languages. For example, the Mississippi Aphasia Screening Test [11] has been adapted for Czech [12], Spanish [13], Persian [14], and Estonian [15]. However, translations of English-language tools often fail to capture essential linguistic, pragmatic, and syntactic features of the target language, thereby limiting their validity in non-English-speaking populations [16]. As such, the development of culturally and linguistically appropriate screening tools is critical for improving diagnostic accuracy and reducing misdiagnosis in people with aphasia [17].

In recent years, several languages have developed their own aphasia screening tools, including the Hungarian Aphasia Screening Test [8] (HAST), the Serbian Aphasia Screening Test [18] (SAST), and the Aphasia Screening Test for European Portuguese [19] (TeRAp), each demonstrating strong reliability, validity, and clinical utility. Specifically, the SAST [18] provides a comprehensive, multimodal evaluation that includes spoken and written language, demonstrating strong reliability and the capacity to differentiate aphasia types, while the HAST [8] offers an efficient, psycholinguistically principled bedside tool with strong diagnostic accuracy and high convergent validity despite its brief administration time. The TeRAp [19] represents a newer generation of digital screening assessments, leveraging automated scoring and remote accessibility while maintaining excellent sensitivity and specificity in identifying aphasia. Together, these instruments highlight current advances in aphasia screening, including improved cultural and linguistic adaptation, enhanced psychometric performance, and the integration of digital technologies. They also reveal persisting gaps such as the variable reporting of reliability indices, differences in modality coverage, and the challenges of balancing test length with diagnostic comprehensiveness. Understanding the characteristics and performance of these tools is critical for guiding clinical decision-making and informing the selection or adaptation of screening instruments within diverse healthcare settings, with an analytic overview of each test presented in Table 1.

However, no brief, locally developed, and psychometrically validated aphasia screening tool is currently available for use in Cyprus. This lack of a culturally and linguistically relevant aphasia screening tool has created a significant gap in Cypriot clinical practice. To address this gap, the Cyprus Aphasia Screening Test (CAST) was developed as a brief and culturally appropriate bedside tool. Taken together, existing aphasia screening tools highlight persistent design tensions, including the trade-off between brevity and diagnostic resolution, limitations in modality coverage, and variable control of psycholinguistic properties. These unresolved challenges informed the design constraints of the CAST.

### 1.2. Development and Main Characteristics of the CAST

The CAST aims to support early identification and referral in Cypriot healthcare settings. The CAST will equip healthcare professionals with a tool to identify aphasia at bedside, minimizing the risk of misdiagnosis or underdiagnosis [6], and will help ensure that Greek-speaking individuals with aphasia in Cyprus receive the necessary treatment within the healthcare system [20].

The development of the CAST was informed by established aphasia screening and assessment practices, drawing conceptually on internationally validated tools: the HAST [8], the SAST [18], and the TeRAp [19], while ensuring linguistic and cultural relevance for Cypriot Greek speakers. The selection of tasks and stimuli was guided by principles commonly applied in aphasia test design, including consideration of psycholinguistic factors known to influence language processing in people with aphasia (PwA) [9]. These factors included lexical frequency, imageability, animacy, word and sentence length, syntactic complexity, and semantic and phonological relationships [8]. Psycholinguistic variables were prioritized based on their established sensitivity to aphasic impairment and feasibility for rapid bedside screening, rather than exhaustive linguistic coverage. The development of the CAST was informed by extensive experience of the research team with Greek adaptations and psychometric validations of aphasia assessment tools used in Cyprus, including the Scenario Test-GR [21], the Aphasia Impact Questionnaire-21-GR [22], and the Communication Effectiveness Index-GR [23].

The CAST development process followed several stages: (1) identifying appropriate subtests and constructing verbal stimuli; (2) creating the administration and scoring procedures; (3) developing the visual materials; (4) conducting pilot testing; and (5) refining items and formatting the final version based on pilot feedback. The resulting screening tool is characterized by the following features: it focuses exclusively on spoken language (both comprehension and production), incorporates stimuli controlled for relevant linguistic and psycholinguistic properties, and uses simple and familiar visual materials [24]. The CAST is designed to be brief and practical for bedside administration, typically requiring less than 10 min to complete. The CAST materials consist of a concise 3-page test form and an attached 1-page form with visual stimuli. Although the CAST is a clinician-administered screening tool rather than a technology-driven intervention, establishing robust, culturally appropriate clinical instruments is a prerequisite for future digital, adaptive, or AI-supported developments in aphasia screening.

### 1.3. Aim

This paper outlines the development of the CAST, detailing its psychometric testing and offering recommendations for clinical use at bedside.

## 2. Materials and Methods

### 2.1. Participants

Three participant groups were recruited for this study: (1) stroke survivors without aphasia (SSwoA), (2) people with aphasia (PwA) following stroke, and (3) healthy controls (HC). Eligibility was determined using predefined inclusion and exclusion criteria. Individuals in the two stroke groups were required to be adults (≥18 years) with a confirmed clinical diagnosis of stroke, while HC were adults with no history of stroke, aphasia, or other neurological conditions. Across all groups, exclusion criteria included a documented diagnosis of dementia, other neurological disorders, clinical depression, or uncorrected visual or hearing impairments that could interfere with task performance or compromise the integrity of the assessment procedures.

Participants with stroke and aphasia were recruited from various regional and national rehabilitation centres, tertiary hospitals, private clinics, and healthcare centres in Limassol, Nicosia, Paphos, Larnaca, and Famagusta districts between February 2024 and February 2025. Healthy controls were recruited via a snowball sampling around the country during the same period.

Ninety-nine people in total participated in the psychometric testing of CAST: 43 were PwA, 21 were SSwoA, and 35 were HC. This sample size is comparable to that of prior reports of validation of aphasia screening tests in other languages [8,18,19]. Details of the demographics of each subgroup are presented in Table 2.

### 2.2. Description of the CAST: Content, Materials, and Scoring

The CAST is a brief language assessment designed for bedside and outpatient use. The CAST is not intended to replace comprehensive language assessment or differential diagnosis (e.g., aphasia versus motor speech disorders), but rather to support rapid identification of aphasia and referral for full speech and language therapy evaluation. Minimal materials are required: a scoring sheet containing a brief case history, scripted instructions, and stimulus prompts; four black-and-white line drawings attached to the scoring sheet for the picture naming task; and four common objects (a door, chair, drinking glass, and tissue) used for the object naming task. Stimuli were selected with reference to lexical frequency, imageability, phonological complexity, and cultural familiarity, following principles commonly applied in aphasia test design. The low-resource design may support feasibility across acute and community clinical environments. The CAST comprises ten subtests that assess aphasia severity, fluency, auditory comprehension, repetition, and naming. Each subtest was selected to maximize sensitivity to core aphasic language processes while maintaining feasibility for rapid bedside administration.

Subtest 1 (Aphasia Severity) involves eliciting a spontaneous speech sample in response to open-ended questions such as “Why are you here?” and “Do you have any concerns about your communication?”. This subtest is intended to provide a global index of expressive language severity and communicative effectiveness, while receptive language abilities are assessed explicitly in subsequent comprehension subtests. Performance is scored on a 6-point scale, where 1 indicates absence of spoken output or profound receptive impairment and 6 reflects minimal language impairment and largely functional communication. The severity score is intended as a global index rather than a weighted composite of individual discourse dimensions.

Subtest 2 (Verbal Fluency) includes a semantic fluency task and a phonemic fluency task. In the semantic task, patients name as many items from a designated semantic category (e.g., animals) as possible within one minute. In the phonemic task, they generate words beginning with a specific letter or phoneme (e.g., /a/). These tasks assess lexical retrieval, search strategy, and executive control. When patients recite over 11 items in a minute, it contributes 1 point.

Subtest 3 (Sentence Completion) requires the patient to provide a single word that meaningfully completes four short sentences read aloud by the examiner. For example, the examiner reads ‘I eat soup with a…’, and the expected response is ‘spoon’. This probes contextual lexical retrieval and sensitivity to syntactic–semantic constraints. Each of the four items is scored dichotomously (1 = correct, 0 = incorrect), based on accurate and contextually appropriate completion.

Subtest 4 (Automatic Speech) evaluates procedural or overlearned language by asking patients to recite well-known sequences such as counting, days of the week, or months of the year. Each sequence is scored for accurate, uninterrupted production. Complete responses receive 1 point; errors or failures receive 0.

Subtest 5 (Yes/No Questions) assesses sentence-level auditory comprehension. The examiner reads four yes/no questions of varying syntactic and semantic complexity (e.g., Do we cut with a spoon?”. Patients respond verbally or by gesture. Each correct response is awarded 1 point, and incorrect or ambiguous responses receive 0.

Subtest 6 (Following Verbal Commands) evaluates comprehension of single-step and multi-step instructions. Patients are asked to carry out commands that increase in length and complexity (e.g., “Show me the spoon, then point to the door”). Accurate execution of each command yields 1 point; incomplete, incorrect, or sequence-violating responses score 0.

Subtest 7 (Word Repetition) consists of four words that increase in phonological and syllabic complexity. The examiner reads each word aloud (e.g., house, chocolate), and the patient repeats it as accurately as possible. Responses are scored dichotomously (1 = phonologically correct repetition, 0 = incorrect), with allowances made only for dialectal or accent-related variations that do not alter phonological structure.

Subtest 8 (Sentence Repetition) requires patients to repeat sentences ranging from short, canonical structures to longer sentences with increased syntactic and semantic complexity (e.g., Help John with algebra). Each repetition is evaluated for reproduction of essential lexical items and overall syntactic structure. Fully correct repetition receives 1 point, and any lexical omissions, substitutions, or major structural distortions receive 0.

Subtest 9 (Object Naming) uses real objects commonly available in clinical settings (e.g., door, chair, drinking glass, tissue). Objects are presented individually, and patients are asked to name each item. Correct naming earns 1 point, while semantic paraphasias, phonological paraphasias, neologisms, or failure to respond receive 0.

Subtest 10 (Picture Naming) employs four black-and-white line drawings affixed to the scoring sheet. The patient is asked to name each picture (ball, knife, toothbrush, hammer), providing an assessment of confrontation naming. Each correct lexical retrieval is scored as 1; incorrect or absent responses score 0.

Across subtests 2–10, scores are summed using a binary scoring system, yielding maximum scores of 10 for fluency, 8 for auditory comprehension, 8 for repetition, and 8 for naming. Binary scoring was adopted to support rapid administration, reduce rater burden, and enhance reliability in acute settings, acknowledging that this approach limits qualitative error analysis. Together with the 6-point Aphasia Severity score, the CAST provides a total possible score of 40 points, with higher scores indicating more preserved language abilities.

### 2.3. Pilot Testing

The initial pilot of the CAST included 15 stroke survivors: 10 people with aphasia (PwA) and 5 stroke survivors without aphasia (SSwoA). The phonemic fluency item (“Tell me as many objects starting with/a/…”) yielded no responses, likely due to the instruction restricting answers to “objects.” The prompt was revised to the standard format (“Tell me as many words starting with/a/…”). A second pilot with 4 PwA and 4 SSwoA showed that the task remained challenging; however, it was retained given its unique contribution to screening, assessing phonological lexical retrieval, supporting differentiation of fluent vs. non-fluent aphasia, engaging executive functions, and complementing semantic fluency. In the Sentence Completion Task, several items produced inconsistent responses. Predictable items (e.g., “Snow has the colour…”) performed well, whereas others elicited culturally influenced alternatives (e.g., “He is cunning like…”, where many participants responded “cat” instead of the target “fox”). Because CAST uses binary scoring, items were revised to reduce variability by selecting prompts with fewer plausible alternatives, such as “I eat soup with a… (spoon)”. A final pilot involving 5 PwA and 5 SSwoA assessed the revised items, administration time, and scoring procedures. The CAST was completed in approximately 8 min, and scores ranged from 7 to 25 for PwA and 36–40 for SSwoA. Pilot testing was intended to assess feasibility, clarity, and administration time rather than to statistically optimize item selection. No further modifications were required.

### 2.4. Main Validation Study

A cross-sectional study was carried out to evaluate the psychometric properties of the CAST. Ethical approval for the study was obtained from the National Bioethics Committee. Informed consent was secured using an accessible, written, signed, and dated form, and demographic information was collected prior to testing. Four qualified Greek-speaking speech and language therapists (SLTs), all working in adult neurorehabilitation, conducted the assessments after receiving training from the study team. The validation of the CAST followed a multi-step process to ensure linguistic and clinical appropriateness for the Greek-speaking population of Cyprus. This included three rounds of pilot testing, as well as assessments of test–retest and inter-rater reliability. Test–retest reliability was evaluated by administering the CAST twice, with a 7–14-day interval, while inter-rater reliability was assessed by comparing results across different SLTs. The test–retest interval ranged from 7 to 14 days to balance the risk of recall effects against the likelihood of clinically meaningful language change. This range reflects pragmatic scheduling constraints typical of clinical and community-based research and is consistent with applied psychometric practice.

Testing was conducted across three visits: the first session (approximately 1 h) included administration of the CAST and the Greek adaptation of the Boston Diagnostic Aphasia Examination Short Form (BDAE-SF) [25]; the second visit (about 8 min) involved re-administration of the CAST for test–retest purposes; and the third visit (also around 8 min) was used to assess inter-rater reliability.

### 2.5. Measures

(i) The Greek adaptation of the Boston Diagnostic Aphasia Examination Short Form (BDAE-SF) [25] served as a valuable reference point for validating the newly developed tool, the CAST, by supporting convergent validity testing. The Greek BDAE-SF [25] measures multiple domains of language function commonly impaired in aphasia, including aphasia severity, spontaneous speech, auditory comprehension, repetition, and naming. Aphasia severity and spontaneous speech are assessed through conversational and expository tasks that examine fluency, grammatical construction, and lexical retrieval. Auditory comprehension is evaluated through tasks that test word discrimination, the ability to follow commands, and the understanding of complex sentences. Repetition tasks involve repeating words, phrases, and sentences, providing insight into phonological processing and verbal working memory. Naming tasks include confrontation and responsive naming, assessing lexical access, and semantic organization. The Greek BDAE-SF takes approximately 45 min to complete. To minimize participant burden and align with the scope of the CAST, only selected BDAE-SF subtests corresponding to CAST domains were administered. These were: the aphasia severity rating, fluency, repetition, auditory comprehension, and naming tasks. As a result, convergent validity claims are restricted to these shared domains.
(ii) The CAST is a brief clinician-reported measure. It consists of a short case history interview followed by ten subtests: a spontaneous speech sample to assess aphasia severity, verbal fluency tasks, auditory comprehension tasks, word and sentence repetition tasks, and object and picture naming tasks. The CAST is scored out of 40, with lower scores warranting a formal clinical speech and language therapy assessment. The CAST is completed in approximately 8 min, depending on language skills.

### 2.6. Data Analysis

Statistical analysis was conducted using JASP [26]. The CAST internal consistency was examined through Cronbach’s α calculations, while intraclass Correlations (ICCs) were implemented to assess test–retest and interrater reliability. To test the CAST’s known-groups validity, one-way ANOVAs were used. Residual normality was visually inspected via Q-Q plots, and variance equality was tested using Levene’s test. Violations of these assumptions were accounted for via the Brown-Forsythe correction. To investigate the validity of the CAST, Spearman correlations were conducted between the CAST scores and the BDAE-SF scores. Finally, to determine the optimal aphasia risk cut-off score, a Receiver Operating Characteristic (ROC) curve was generated using aphasia diagnosis as the point of reference.

To evaluate the reliability of the CAST, several Psychometric criteria were examined:

- Internal consistency was assessed to determine the extent to which test items measured a unified construct, with Cronbach’s α values above 0.70 considered acceptable [27] and values at or above 0.80 regarded as excellent, following standards used in other aphasia screening tools [8].
- Test–retest reliability was examined to determine the temporal stability of CAST scores, with intraclass correlation coefficients (ICCs) of 0.75 or higher indicating good reliability [28].
- Interrater reliability was assessed using ICCs as well, with coefficients of 0.80 or above interpreted as evidence of strong agreement between independent raters [28]. High test–retest and interrater reliability together ensure that the CAST produces consistent and reproducible results across time and examiners.

Multiple forms of validity were assessed to determine whether the CAST accurately identifies aphasia:

- Convergent validity was evaluated using correlations between CAST performance and equivalent subtests from the Greek version of the BDAE-SF, following procedures used in comparable validation studies [8]. Correlation strength was interpreted according to established psychometric thresholds: weak (<0.30), moderate (0.40–0.60), and strong (>0.60) [29].
- Known-groups validity was examined by testing whether the CAST could distinguish among people with aphasia, stroke survivors without aphasia, and healthy controls; statistically significant group differences (with α = 0.05) were taken as evidence of valid discrimination [30].
- Diagnostic validity was assessed using the Area Under the Curve (AUC) metric, with AUC values of 0.80 or higher considered clinically useful, values near 1.0 indicating excellent discrimination, and values near 0.50 reflecting chance-level classification [28]. Optimal cut-off scores were determined by identifying the point at which sensitivity and specificity were maximized [31].

## 3. Results

Descriptive statistics (means, standard deviations, and score ranges) for the CAST total score, CAST subtests, and reference measures are presented for people with aphasia (PwA), stroke survivors without aphasia (SSwoA), and healthy controls (HC) in Table 3.

### 3.1. Internal Consistency

Internal consistency of the CAST was examined through Cronbach α calculations. Overall, the CAST demonstrated excellent internal consistency (α = 0.967, 95% CI = [0.960, 973]). Further explorations, through item-rest correlations, indicated that CAST item 2.2 performed poorly (α = 0.171) compared to all other items, which ranged between α = 0.411–0.907. Item 2.2 involved a phonemic fluency task where participants were asked to name as many words as they could beginning with/a/in 1 min. They were given 1 point when they retrieved more than 11 words.

### 3.2. Intraclass Correlations

ICCs were implemented to assess test–retest reliability, as well as interrater reliability of the CAST. Both test–retest (ICC = 0.983, 95% CI = [0.974, 0.988]) and interrater (ICC = 0.979, 95% CI = [0.969, 0.986]) results revealed excellent reliability.

### 3.3. Known Groups Validity

**Figure 1:**
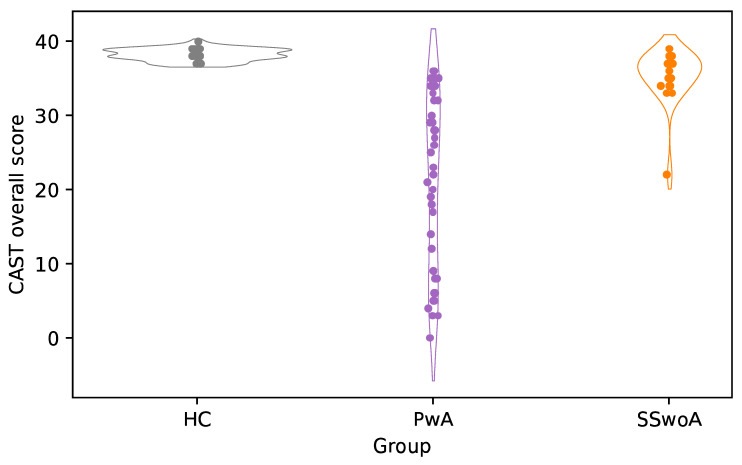
Individual CAST scores across the three groups. Notes: PwA, People with Aphasia; SSwoA, Stroke Survivors without Aphasia; HC, Healthy Controls.

To test the CAST’s known groups validity, one-way ANOVAs were used to test CAST score differences across the three groups (PwA vs. SSwoA vs. HC). As illustrated in Figure 1, differences were evident across the three groups for the overall CAST scores (F_(2,51_._57)_ = 70.068, *p* < 0.001).

Tuckey corrected post hoc *t*-test further showed that these group differences derived from the lower scores of PwA compared to both HC (*t*_(96)_ = −9.287, *p* < 0.001) and SSwoA (t_(96)_ = −6.666, *p* < 0.001). No differences were found between HC and SSwoA (t_(96)_ = −1.231, *p* = 0.438). Notably, a similar pattern of results was observed across all CAST domains; detailed domain analyses are presented in Table 4.

### 3.4. Validity Analyses

**Figure 2:**
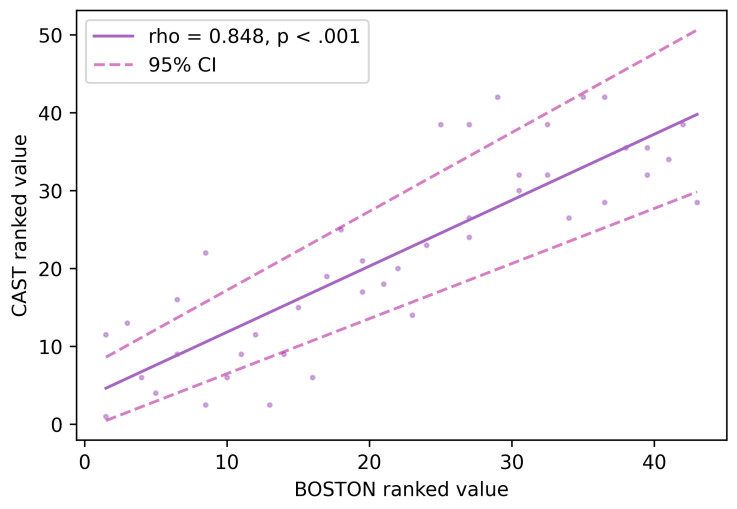
CAST-BDAE-SF total score correlation analysis. Notes: CAST, Cyprus Aphasia Screening Test; BDAE-SF, Boston Diagnostic Aphasia Examination Short Form.

To investigate the validity of the CAST, Spearman correlations were conducted between the CAST scores and the BDAE-SF scores. A significant correlation analysis between the total scores of the CAST and the BDAE-SF confirmed a linear relationship across the two measures (ρ = 0.848, *p* < 0.001). See Figure 2.

Similarly, high correlational values were found when testing each of the five domains of the two measures. The details of the correlation results across domains are provided in Table 5.

### 3.5. Cut-Off Scores

**Figure 3:**
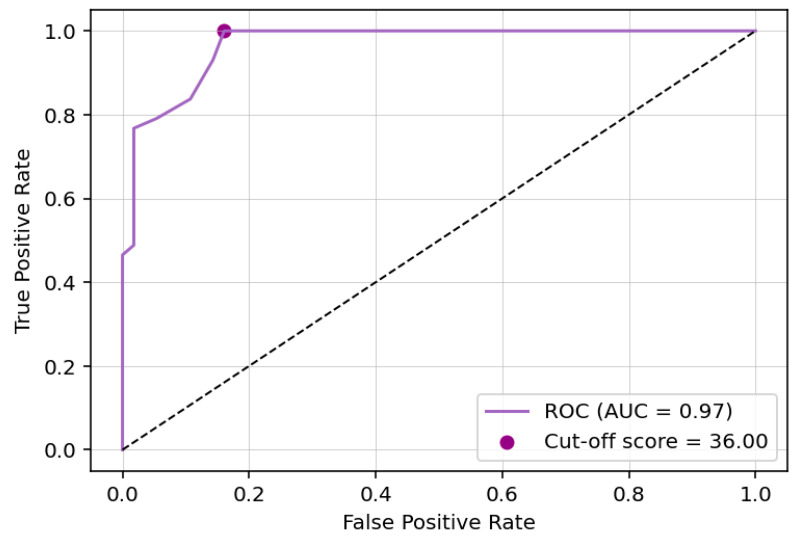
CAST cut-off score ROC curve. Notes: CAST, Cyprus Aphasia Screening Test; ROC, Receiver Operating Characteristic.

To calculate aphasia ‘at risk’ cut-off scores, a Receiver Operating Characteristic (ROC) curve was fitted, based on aphasia diagnosis. The ROC curve (Figure 3) showed excellent discriminatory ability (area under the curve = 0.97, 95% CI = [0.94, 0.99]), identifying a CAST score of 36 as the optimal cut-off (Sensitivity = 1.00, 95% CI = [0.82, 1]; Specificity = 0.84, 95% CI = [0.76, 1]).

To further validate this cut-off score, we calculated the 90th percentile of the PwA group CAST score as an upper bound of aphasia risk, and the 10th percentile of the control group CAST score as a lower bound of no aphasia risk. The optimal cut-off score fell between these bounds (lower, upper = [35, 37]), supporting the results obtained from the ROC analysis. Percentile details for all three groups are presented in Table 6.

**Figure 4:**
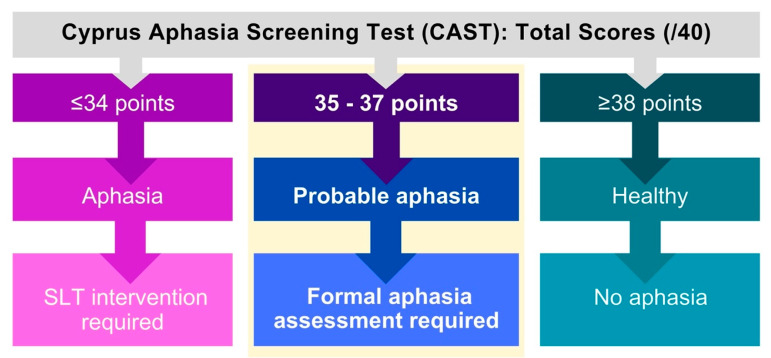
Decision tree for interpreting CAST total scores. Note: SLT, Speech and Language Therapy.

See Figure 4 for interpreting total CAST scores.

## 4. Discussion

This paper reports the development and validation of the CAST, the first aphasia screening test in Cyprus. It comprises 10 subtests, that screen speech comprehension and production, and is designed to be easily administered and scored at bedside.

The creation of the CAST addresses the absence of a standardized aphasia screening tool for Greek-speaking populations in Cyprus. It provides cultural and linguistic localization, aligned with contemporary psychometric validation standards, to support the identification of language impairments and strengthen clinical aphasia assessment practices across the country.

### 4.1. Overall Reliability

Internal consistency analysis yielded excellent results, suggesting that the CAST tasks reliably assess a unified underlying construct, language impairment. The high internal consistency observed likely reflects the shared influence of overall aphasia severity across language domains rather than strict unidimensionality. As a screening tool, the CAST is intended to capture multiple related aspects of language impairment, and factors such as binary scoring and inter-item overlap may contribute to elevated alpha values. Most individual items correlated well with the overall scale, with only one lower-performing item (item 2.2 phonemic fluency task). This low item-rest correlation suggests that this fluency task is poorly correlated with the overall test performance [32]. Several factors may explain this outcome, including the simplicity of the scoring method, which may have reduced sensitivity. Specifically, participants received 1 point only if they produced more than 11 words within one minute- a coarse, binary scoring approach. This scoring approach also reduces the variability in responses and makes it less sensitive to individual differences, thereby weakening its statistical relationship with other items. Also, the task’s difficulty might have influenced the performance of the specific item, as phonemic fluency tasks are cognitively demanding as they draw not only on language but also executive functions (e.g., strategic search, inhibition of repeats), working memory, and speed of processing [33]. As the rest of the CAST focuses more on core language functions (e.g., naming, repetition, auditory comprehension), this phonemic fluency task taps into different ‘executive’ aspects of verbal behaviour, such as accessing lexical elements, thinking flexibly, and switching between response sets, or the ability to self-regulate and self-monitor [32].

What is interesting to note here is that 77% of the people with aphasia who participated in this study had left hemispheric lesions, which are associated with significant impairments in phonological fluency when compared to semantic fluency [34]. Furthermore, in Greek, phonemic fluency tasks are considered less ecologically valid than semantic fluency tasks (e.g., naming animals), as cultural and educational familiarity with phonemic tasks can vary significantly. This variability may influence performance independently of aphasia severity [35]. Further, floor effects may have occurred, as it is likely that many participants with aphasia scored zero, especially since the majority experienced moderate-to-severe naming symptoms, resulting in limited variance and a skewed distribution [36]. These interpretations are speculative and were not empirically tested within the present study. They should therefore be considered hypotheses for future research rather than explanatory conclusions. Finally, test–retest and interrater reliability were high, suggesting that CAST scores are stable over time and robust across different raters.

### 4.2. Overall Validity

The CAST demonstrated strong known-groups validity by significantly distinguishing between PwA, SSwoA, and HC. PwA scored consistently lower across all language domains (fluency, comprehension, repetition, and naming), highlighting the tool’s sensitivity to language impairments specific to aphasia. In contrast, no significant differences were observed between SSwoA and HC, confirming that CAST is specific to aphasia-related deficits rather than general effects of stroke. This supports the CAST’s clinical utility and specificity in identifying aphasia.

Convergent validity was supported by strong correlations between CAST scores and those of the Greek BDAE-SF in all corresponding domains (aphasia severity, fluency, repetition, comprehension, naming). The overall correlation between the two tools confirms that CAST measures equivalent constructs to a widely used gold standard, reinforcing its diagnostic credibility. Nevertheless, these findings should be interpreted within the limits of partial reference test administration.

### 4.3. Comparison of the CAST with Other Published Screening Tools

The CAST supports psychometric properties that compare favourably with other recently developed non-English aphasia screening tools, including the Hungarian Aphasia Screening Test [8] (HAST), the Serbian Aphasia Screening Test [18] (SAST), and the Aphasia Screening Test for European Portuguese [19] (TeRAp). Regarding internal consistency, the CAST exhibited excellent reliability, marginally exceeding the corresponding values reported for HAST, SAST, and TeRAp. Test–retest and interrater reliability were also highest for the CAST across the other tools. All four instruments demonstrated robust known-groups validity, effectively distinguishing individuals with aphasia from both stroke patients without aphasia and neurologically healthy controls. Furthermore, the CAST also demonstrated strong convergent validity with the Greek version of the BDAE-SF, with correlation coefficients similar to those reported for the HAST with the Hungarian version of the BDAE-HU, the SAST with the Serbian Western Aphasia Battery, and TeRAp with the Lisbon Aphasia Assessment Battery. For a detailed comparison of psychometric indices, see Table 7.

### 4.4. CAST Cut-Off Score

The CAST includes a standardized severity threshold of 36/40, indicating that scores below 36 may reflect the presence of aphasia. This cut-off score serves as a clinically meaningful benchmark, guiding professionals in identifying individuals who may require further in-depth formal language assessment and/or intervention [31]. By offering a standardized reference point, the CAST facilitates consistent interpretation across clinical settings and supports informed decision-making for referral planning.

Nevertheless, the proposed cut-off score should be considered preliminary and requires confirmation in larger, independent samples before routine clinical implementation. While the ROC analysis indicated excellent discrimination, the cut-off score derived within the same sample may yield optimistic estimates. Sensitivity–specificity trade-offs should therefore be considered in relation to screening priorities rather than diagnostic certainty.

### 4.5. Clinical Implications

In clinical practice, the CAST can differentiate stroke patients with and without aphasia, enabling timely and appropriate referrals to speech and language therapy. Early detection of aphasia is critical for initiating targeted interventions that support functional communication, participation in everyday life, and long-term recovery [37]. By facilitating early aphasia identification, the CAST plays a key role as a screening measure in reducing the risk of long-term communication barriers after stroke and supporting rehabilitation outcomes for both the individual and their care environment [38].

With appropriate training and implementation evaluation, the CAST may have potential for use by healthcare professionals beyond speech and language therapists. In stroke units and rehabilitation settings in Cyprus, it can be administered by nurses, occupational therapists, physiotherapists, and other allied health professionals as part of routine interdisciplinary screening of stroke symptoms [20]. Additionally, physicians in hospital follow-up clinics or private practice can use the CAST to support initial screening and guide timely SLT referrals. This flexibility supports seamless integration into routine clinical workflows and increases the likelihood of early identification of individuals with aphasia [39].

The standardization of the CAST lays the groundwork for a more comprehensive, culturally appropriate aphasia assessment framework tailored to the needs of the Greek-speaking population in Cyprus. Beyond its local utility, the CAST contributes to the global landscape of aphasia screening by aligning with international best practices in the detection of language impairments after stroke.

### 4.6. Limitations

Despite its promise, the CAST is subject to several limitations that warrant consideration. First, the study included only adults with post-stroke aphasia; therefore, its applicability to other aetiologies of aphasia remains untested. Second, although efforts were made to ensure dialect neutrality, individual linguistic variation within Greek-Cypriot participants and across educational backgrounds may still influence test performance, particularly among older adults or those with limited literacy. Third, the recruitment of individuals in the hyperacute phase (0–3 months post-stroke) was not feasible due to restricted access to government stroke units. Fourth, the healthy control group was younger than both stroke groups, and although the primary contrast of interest (people with aphasia versus stroke survivors without aphasia) involved age-comparable groups, age-related effects on language performance cannot be entirely ruled out. Future studies with larger samples should incorporate age-matched controls or age-adjusted analyses. Also, participant characterization was limited by the availability of clinical data across recruitment sites. Although time post-stroke was reported and comparable between stroke groups, information on aphasia subtype, detailed lesion characteristics, and neuroimaging data was not consistently available. This limits a more fine-grained interpretation of the psychometric findings and subgroup analyses. Future validation studies should aim to incorporate more comprehensive clinical profiling to support construct-level interpretation of screening performance. Additionally, the assessors were not blinded to the diagnostic group, which reflects real-world clinical practice but may introduce bias. Additionally, ceiling effects and restricted score ranges were observed in the stroke-without-aphasia and healthy control groups. These characteristics may inflate interrater and test–retest reliability estimates and limit the interpretability of subgroup-specific reliability analyses. Additional limitations include potential item redundancy due to overlapping task demands and restricted variance in control group scores. These factors should be considered when interpreting reliability and diagnostic accuracy estimates. Moreover, handedness was not systematically documented, limiting conclusions regarding crossed aphasia in participants with right-hemisphere lesions. Finally, most participants in the two experimental groups were approximately 18 months post-stroke, placing them in the chronic phase. This may have affected performance, as people with chronic aphasia often develop compensatory communication strategies to overcome verbal deficits [40].

### 4.7. Future Directions

Future research should consider expanding the CAST’s validation to a larger and more diverse sample across different healthcare contexts (e.g., national stroke units). Also, future studies with larger samples could explore the underlying factor structure of the CAST using appropriate psychometric models. Also, the selected cut-off score prioritizes sensitivity, which is appropriate for screening contexts but may increase false-positive referrals. Alternative cut-off points and their clinical implications should be explored in future studies with larger and independent samples. Additionally, the potential for digitizing the CAST offers an avenue for remote screening, especially in rural or underserved regions of the country. Cross-cultural adaptations could also explore the CAST’s applicability in Greek-speaking populations outside Cyprus, particularly in Greece. Future refinements of the CAST could also incorporate structured content-validation methods, such as questionnaire-based feedback from multidisciplinary clinicians (e.g., neurologists, nurses, and rehabilitation professionals), to further strengthen content validity. Finally, incorporating patient and public involvement [41] in future refinements could increase the usability and acceptability of the content from the perspective of people with aphasia.

## 5. Conclusions

The development of the CAST marks a significant advancement in aphasia screening for the Greek-speaking population in Cyprus. By providing a standardized, validated screening tool, the CAST addresses the pressing need for a relevant aphasia screening tool for Cyprus. The CAST can be used to improve aphasia detection in clinical settings, improving the referral path to comprehensive SLT assessments and more timely diagnosis, resulting in targeted rehabilitation and improved outcomes in Greek-Cypriot people with aphasia.

## Figures and Tables

**Table 1:** Comparison of SAST, HAST, and TeRAp Aphasia Screening Tests.

Screening Test	HAST	SAST	TeRAp
Origin	Hungary	Serbia	Portugal (as a digital app).
Administration time	5–10 min	10–20 min	Rapid digital screener (time not explicitly stated).
Total Subtests	5: (1) Word comprehension, (2) Sentence comprehension, (3) Repetition, (4) Naming, (5) Word fluency.	10 + 1 unscored: (1) Automatized sequences, (2) Auditory comprehension, (3) Visual confrontation naming, (4) Responsive naming, (5) Word repetition, (6) Sentence repetition; (7) Word reading, (8) Oral sentence reading, (9) Reading comprehension, (10) Writing, (11) Conversation (used for clinical impression, not scored).	6: (1) Speech description, (2) Naming, (3) Auditory comprehension, (4) Repetition, (5) Reading aloud, (6) Writing.
Sample size (validation)	117 (40 PWA; 26 strokes without aphasia; 51 controls).	240 (120 PWA; 120 controls)	475 total (257 healthy; 218 clinical: aphasia, dysarthria, MCI)
Internal Consistency	α = 0.74	a = 0.98	α = 0.99
Inter-rater Reliability	Not reported	ICC = 0.94	r = 0.96
Test–retest Reliability	Not reported	r = 0.74	r = 0.96
Convergent Validity	ρ = 0.81 Strong correlation with WAB	ρ = 0.87 Strong correlation with BDAE	ρ = 0.78 Strong correlation with BAAL
Diagnostic Accuracy: AUC	AUC = 0.95 (95% CI [0.89–1.00])	Not reported	Not reported
Diagnostic Accuracy: Sensitivity	92.5%	Not reported	1.00 (100%)
Diagnostic Accuracy: Specificity	Stroke controls: 88.5% Healthy controls: 96.1%	Not reported	0.99 (99%)
ROC Cut-off	≤17	Not reported	Not provided (algorithm-based scoring)
Effect Sizes	Group differences reported but no d or η^2^	Subtest Cohen’s d values: 0.79–6.07	Not reported
Clinical Strengths	Very brief; high diagnostic accuracy; bedside-appropriate; controlled stimuli.	Comprehensive multimodality assessment; sensitive to aphasia type and severity; large sample size.	Digital; automatic scoring; high sensitivity/specificity; wide modality coverage.
Limitations	Few items; lacks reading/writing; some reliability indices missing.	Longer, literacy-dependent tasks may challenge acute/severe cases.	Requires device/internet; some psychometrics not reported.

Note: SAST, Serbian Aphasia Screening Test; HAST, Hungarian Aphasia Screening Test; TeRAp, The Aphasia Screening Test; BDAE, Boston Diagnostic Aphasia Examination; PWA, People with Aphasia; WAB, Western Aphasia Battery; BAAL, Lisbon Aphasia Assessment Battery.

**Table 2:** Demographic data of the participants by group.

Characteristic	PwA (*n* = 43)	SSwoA (*n* = 21)	Controls (*n* = 35 *)	Subgroup Differences
*Sex*				
Male	23 (53%)	14 (67%)	10 (29%)	χ^2^_(4)_ = 10.75 *p* = 0.03
Female	20 (47%)	7 (33%)	22 (65%)	
Other	-	-	2 (6%)	
*Age*				
Mean (sd)	68.51 (12.09)	64.71 (13.89)	49.47 (7.20)	*F*_(2, 95)_ = 29.35 *p <* 0.001
Min-Max	35–90	32–89	41–69	
*Stroke Type*				
Ischemic	30 (70%)	17 (81%)	N/A	χ^2^_(1)_ = 0.95 *p* = 0.34
Haemorrhagic	13 (30%)	4 (19%)	N/A	
*Lesion Side*				
Left	33 (77%)	9 (43%)	N/A	χ^2^_(1)_ = 7.18 *p* = 0.007
Right	10 (23%)	12 (57%)	N/A	
*Hemiplegia*				
Left	9 (21%)	8 (38%)	N/A	χ^2^_(1)_ = 4.31 *p* = 0.12
Right	30 (70%)	9 (43%)	N/A	
None	4 (9%)	4 (19%)	N/A	
*Months post stroke diagnosis*				
Mean (sd)	18.54 (22.10)	17.95 (20.84)	N/A	*t*_(62)_ = 0.10 *p* = 0.92
Min–Max	3–96	3–82	N/A	
*Completed education*				
Elementary School	9 (21%)	3 (14%)	2 (6%)	χ^2^_(10)_ = 28.60 *p* = 0.001
High School	20 (47%)	12 (57%)	17 (50%)	
College	-	3 (14%)	8 (24%)	
Bachelor’s	13 (30%)	-	6 (18%)	
Master’s	1 (2%)	3 (14%)	-	
Other	-	-	1 (3%)	
*Marital Status*				
Married	35 (81%)	15 (71%)	27 (79%)	χ^2^_(1)_ = 20.71 *p* = 0.02
Divorced	4 (9%)	1 (5%)	4 (12%)	
Widowed	2 (5%)	-	-	
Single	2 (5%)	4 (19%)	-	
Engaged	-	1 (5%)	-	
Other	-	-	3 (9%)	

Notes. N/A, not applicable; PwA, People with Aphasia; SSwoA, Stroke Survivors without Aphasia; HC, Healthy Controls; CAST = Cyprus Aphasia Screening Test. * Missing data from one participant.

**Table 3:** Test scores by group.

Test	Group	Mean	SD	Minimum	Max
CAST-Overall	PwA	20.95	12.11	0	36
	SSwoA	35.48	3.54	22	39
	HC	38.26	0.82	37	40
CAST-ASRS	PwA	3.12	1.50	1	5
	SSwoA	5.67	0.91	2	6
	HC	6	0	6	6
CAST-Fluency	PwA	3.47	2.67	0	8
	SSwoA	7.14	1.71	3	10
	HC	8.46	0.70	7	10
CAST-Auditory Comprehension	PwA	5.37	2.68	0	8
	SSwoA	7.71	0.46	7	8
	HC	7.94	0.24	7	8
CAST-Repetition	PwA	4.30	3.23	0	8
	SSwoA	7.62	0.97	4	8
	HC	7.91	0.28	7	8
CAST-Naming	PwA	4.09	3.32	0	8
	SSwoA	7.71	0.56	6	8
	HC	7.94	0.24	7	8
BDAE-SF—Total	PwA	36.651	24.217	0	68
BDAE-SF—ASRS	PwA	2.49	1.58	0	5
BDAE-SF—Fluency	PwA	2.36	1.81	0	4
BDAE-SF—Auditory Comprehension	PwA	16.81	10.45	0	32
BDAE-SF—Repetition	PwA	3.98	3.06	0	7
BDAE-SF—Naming	PwA	10.49	8.62	0	23

Notes: PwA, People with Aphasia; SSwoA, Stroke Survivors without Aphasia; HC, Healthy Controls; CAST, Cyprus Aphasia Screening Test; ASRS, Aphasia Severity Rating Scale; BDAE-SF, Boston Diagnostic Aphasia Examination Short Form.

**Table 4:** Domain known- groups validity results for the three groups.

Subcategory	*F*-Test (Brown-Forsythe Corrected)	*t*-Test (Tuckey Corrected)	
ASRS	*F*_(2,61.91)_ = 96.759, *p* < 0.001	PwA vs. HC	*t*_(96)_ = −11.776, *p* < 0.001
		PwA vs. SSwoA	*t*_(96)_ = −8.906, *p* < 0.001
		HC vs. SSwoA	*t*_(96)_ = 1.123, *p* = 0.503
Fluency	*F*_(2,67.52)_ = 77.478, *p* < 0.001	PwA vs. HC	*t*_(96)_ = −11.110, *p* < 0.001
		PwA vs. SSwoA	*t*_(96)_ = −6.999, *p* < 0.001
		HC vs. SSwoA	*t*_(96)_ = 2.412, *p* = 0.046
Auditory Comprehension	*F*_(2,46.16)_ = 35.316, *p* < 0.001	PwA vs. HC	*t*_(96)_ = −6.302, *p* < 0.001
		PwA vs. SSwoA	*t*_(96)_ = −4.910, *p* < 0.001
		HC vs. SSwoA	*t*_(96)_ = 0.462, *p* = 0.889
Repetition	*F*_(2,52.35)_ = 44.593, *p* < 0.001	PwA vs. HC	*t*_(96)_ = −7.242, *p* < 0.001
		PwA vs. SSwoA	*t*_(96)_ = −5.687, *p* < 0.001
		HC vs. SSwoA	*t*_(96)_ = 0.488, *p* = 0.877
Naming	*F*_(2,45.75)_ = 52.900, *p* < 0.001	PwA vs. HC	*t*_(96)_ = −7.628, *p* < 0.001
		PwA vs. SSwoA	*t*_(96)_ = −6.136, *p* < 0.001
		HC vs. SSwoA	*t*_(96)_ = 0.374, *p* = 0.926

Notes: ASRS, Aphasia Severity Rating Scale.

**Table 5:** Domain correlations between CAST and BDAE-SF.

Subgroup	Spearman ρ	*p*-Value
ASRS	0.890	<0.001
Fluency	0.860	<0.001
Auditory Comprehension	0.782	<0.001
Repetition	0.740	<0.001
Naming	0.855	<0.001

Notes: CAST, Cyprus Aphasia Screening Test; BDAE-SF, Boston Diagnostic Aphasia Examination Short Form; ASRS, Aphasia Severity Rating.

**Table 6:** CAST score percentiles per group.

	*Percentile*				
Group	10th	25th	50th	75th	90th
PwA	5	8	23	32	35
SSwoA	33	34	37	37	38
HC	37	38	38	39	39

Notes: CAST, Cyprus Aphasia Screening Test; PwA, People with Aphasia; SSwoA, Stroke Survivors without Aphasia; HC, Health Controls.

**Table 7:** Comparison of CAST, HAST, SAST, and TeRap psychometric indices.

Tool	Internal Consistency (Cronbach’s α)	Test–Retest Reliability (ICC)	Interrater Reliability (ICC)	Known-Groups Validity	Convergent Validity
CAST (Charalambous et al., 2025 [20])	α = 0.96	ICC = 0.98	ICC = 0.97	Significant group differences across PwA, SSwoA, and HC (*p* < 0.001)	ρ = 0.84 with BDAE-SF
HAST (Zakariás & Lukács, 2023 [8])	α = 0.74	Not reported	Not reported	PwA vs. non-aphasic stroke vs. controls, (all *p* < 0.001)	ρ = 0.81 with BDAE-HU
SAST (Vuković et al., 2024 [18])	α = 0.98	ICC > 0.74	ICC = 0.94	Strong separation among clinical groups (*p* < 0.001)	ρ = 0.87 with Serbian version of WAB
TeRAp (Fonseca et al., 2024 [19])	α = 0.99	ICC = 0.96	ICC = 0.91	PwA scored significantly lower than stroke and control groups (*p* < 0.001)	ρ = 0.78 with BAAL

Note: CAST, Cyprus Aphasia Screening Test; HAST, Hungarian Aphasia Screening Test; SAST, Serbian Aphasia Screening Test; TeRAp, The Aphasia Screening Test; BDAE, Boston Diagnostic Aphasia Examination; WAB, Western Aphasia Battery; BAAL, Lisbon Aphasia Assessment Battery.

## Data Availability

Data generated during the current study and supporting the conclusions of this article are included in the manuscript, with any additional data requests to be directed to the corresponding author for consideration.

## Abbreviations

The following abbreviations are used in this manuscript:

CAST	Cyprus Aphasia Screening Test
ASRS	Aphasia Severity Rating Scale
BDAE-SF	Boston Diagnostic Aphasia Examination Short Form
PwA	People with Aphasia
HC	Healthy Controls
SSwoA	Stroke Survivors without Aphasia
HAST	Hungarian Aphasia Screening Test
SAST	Serbian Aphasia Screening Test
TeRAp	Aphasia Screening Test for European Portuguese
